# Mr. Teale’s Case of High Temperature

**Published:** 1875-10

**Authors:** 


Mr. Teale's Case of High Temperature.—Although five months have elapsed since notes of a remarkable case of high temperature were read before the Clinical Society by Mr. Teale, (see July issue of this Journal, page 320), yet the case was in itself so subversive of all current ideas on the subject that we doubt not it is still fresh in the memory of our readers. With commendable candour the author of that paper has just addressed a letter to a contemporary in which he replies *seriatim* to several very natural queries that have been put to him concerning the facts of the case, and the possibilities of any artificial means within reach of the patient of raising the index of the thermometer. From this it would appear that observations were taken in mouth and rectum as well as in the axillæ, with the result of showing the same high level (110° to 120°); that the rise in the thermometer was sometimes noticed at the time: that no hot bottles or blankets were in contact with the patient by which she could have artificially warmed the thermometer; and that the instruments used had been tested and found correct, and had during the recovery of the patient registered normal as well as abnormal temperatures. As we ventured at the time to question the possibility of the maintenance of life at such a high rate of temperature (unaccompanied as it was by any extreme variation in pulse and respiration frequency), so we do now cheerfully accept Mr. Teale's further explanations, which seem to leave nothing to be desired in testimony of the faithfulness and accuracy of the record; while at the same time it leaves the *modus operandi* in as mysterious a condition as ever, and goes far to expand our hitherto too narrow notions. Exceptional and full of mystery the case must remain, and that another of like nature will occur is to be hoped, lest we may have to consign it to that limbo of "exceptions" which go to prove the rule, the rule in this instance being the incompatibility of animal life with a body heat of 120°.—*Lancet, July* 17, 1875.—*Monthly Abstract.*

